# Editorial: Crosstalk between cancer-associated fibroblasts and tumor cells in the tumor microenvironment: an emerging target of anti-cancer immunotherapy

**DOI:** 10.3389/fphar.2023.1256643

**Published:** 2023-08-04

**Authors:** Chunxue Zhang, Runbin Sun, Bo Kong, Rong Hu, Qianming Du

**Affiliations:** ^1^ School of Basic Medicine and Clinical Pharmacy, China Pharmaceutical University, Nanjing, China; ^2^ Phase I Clinical Trials Unit, Nanjing Drum Tower Hospital, Affiliated Hospital of Medical School, Nanjing University, Nanjing, China; ^3^ Nanjing Drum Tower Hospital, Clinical College of Nanjing, University of Chinese Medicine, Nanjing, China; ^4^ Department of Pharmacology and Toxicology, Rutgers, The State University of New Jersey, Piscataway, NJ, United States; ^5^ State Key Laboratory of Natural Medicines, Department of Physiology, China Pharmaceutical University, Nanjing, Jiangsu, China; ^6^ General Clinical Research Center, Nanjing First Hospital, Nanjing Medical University, Nanjing, China

**Keywords:** tumor microenvironment (TME), cancer-associated fibroblasts (CAFs), immune cells, CAF-targeted therapy, cancer

Extensive researches have been conducted on Cancer Associated Fibroblasts (CAFs) during the past several years. As a key component of the tumor microenvironment with crucial functions, CAFs regulate tumor progression, malignant transformation and immune response by interacting with tumor cells and the other cellular components. Extensive studies on CAFs will help to discover new targets for individualized treatments.

This Research Topic, titled “*Crosstalk Between Cancer-Associated Fibroblasts and Tumor Cells in the Tumor Microenvironment: An Emerging Target of Anti-cancer Immunotherapy*,” focuses on the roles of specific subpopulations of CAFs in different types of tumors. Although most studies have highlighted the tumor-promoting effects of CAFs in this Research Topic, it is worth to note that CAFs exhibit various phenotypes and functions in different tumor types and individuals. Further investigations on these subgroup identification and functional characterization, and the associated potential therapeutic targets will benefit the development of targeted therapeutic strategies and the efficacy of cancer treatment. In this Research Topic, Zhang et al. conducted a comprehensive analysis by integrating multiple single-cell RNA sequencing (scRNA-seq) datasets from various tumors and adjacent normal tissues, which discovered that COL11A1^+^ fibroblasts only present in tumor tissue but not normal tissue fibroblasts. These fibroblasts might promote tumor progression by regulating extracellular matrix (ECM) remodeling and anti-tumor immune response (Zhang et al.). Research has shown that CAFs exhibit multiple functions in epithelial ovarian cancer and are closely related to tumor progression. Zou et al. identified multiple subtypes of CAFs through extensive tumor data analysis and found that these subtypes are closely related to the clinical outcomes and immunotherapy response of epithelial ovarian cancer. This breakthrough enhances the understanding of CAF heterogeneity in ovarian cancer and provides guidance for personalized treatment (Zou et al.). In addition, Zhihao et al. revealed that there are differences in the expression of CAFs subtypes and pathways in Osteosarcoma, and these differences are related to tumor progression and immunosuppression (Zhihao et al.). These studies expand our understanding of the tumor diversity and complexity, and provide new insights into specific CAF subgroups. Through the integration of data from multiple cancer types, researchers discovered that NT5E is associated with upregulated expression of CAFs in various cancers, potentially serving as a novel prognostic biomarker linked to specific tumor microenvironments. The increased expression of NT5E may be related to enhanced epithelial-mesenchymal transition (EMT) function in CAFs, presenting new possibilities for immunotherapy by targeting CAFs with high NT5E expression (Xue et al.). Similarly, in gastrointestinal cancer, RAB6B shows promise as a prognostic marker associated with alterations in the tumor immune microenvironment (Peng et al.). These findings contribute to the identification of new prognostic markers crucial for assessing tumor prognosis and individualized treatment.

This Research Topic also covers recent advances in the functions of CAFs’ in tumor progression and treatment. CAFs influence tumor cell proliferation, migration, and invasive capabilities through secreted growth factors, extracellular matrix remodeling, and immune regulation (Wu et al.). For instance, CAFs derived from cholangiocarcinoma (CCA) promote cell viability and enhance gemcitabine resistance in CCA cells by activating IL-6/STAT3 signaling. Inhibition of IL-6R on CCA cells using tocilizumab, an IL-6R humanized antihuman monoclonal antibody, impedes the CAF-CCA interaction, leading to increased gemcitabine sensitivity in CCA cells (Kittirat et al.). Halofuginone inhibits migration and invasion of oral squamous cell carcinoma (OSCC) by targeting CAFs, reducing the malignancy of CAFs, and decreasing the viability and proliferation of OSCC-derived CAFs (Wang et al.).

Current advances regarding the efficacy of immunotherapy by targeting CAFs are also included in this Research Topic. A systematic review and meta-analysis demonstrated the effectiveness of PD-1 inhibitors in treating recurrent/metastatic nasopharyngeal carcinoma, particularly in patients with CAF infiltration, suggesting the influence of CAFs on immunotherapy (Yan et al.). The overview of possible therapeutic strategies including inhibition of CAFs’ immunomodulatory function, suppression of immunosuppressive factors produced by CAFs, and modification of the extracellular matrix secreted by CAFs are discussed by Zhang et al.

**FIGURE 1:**
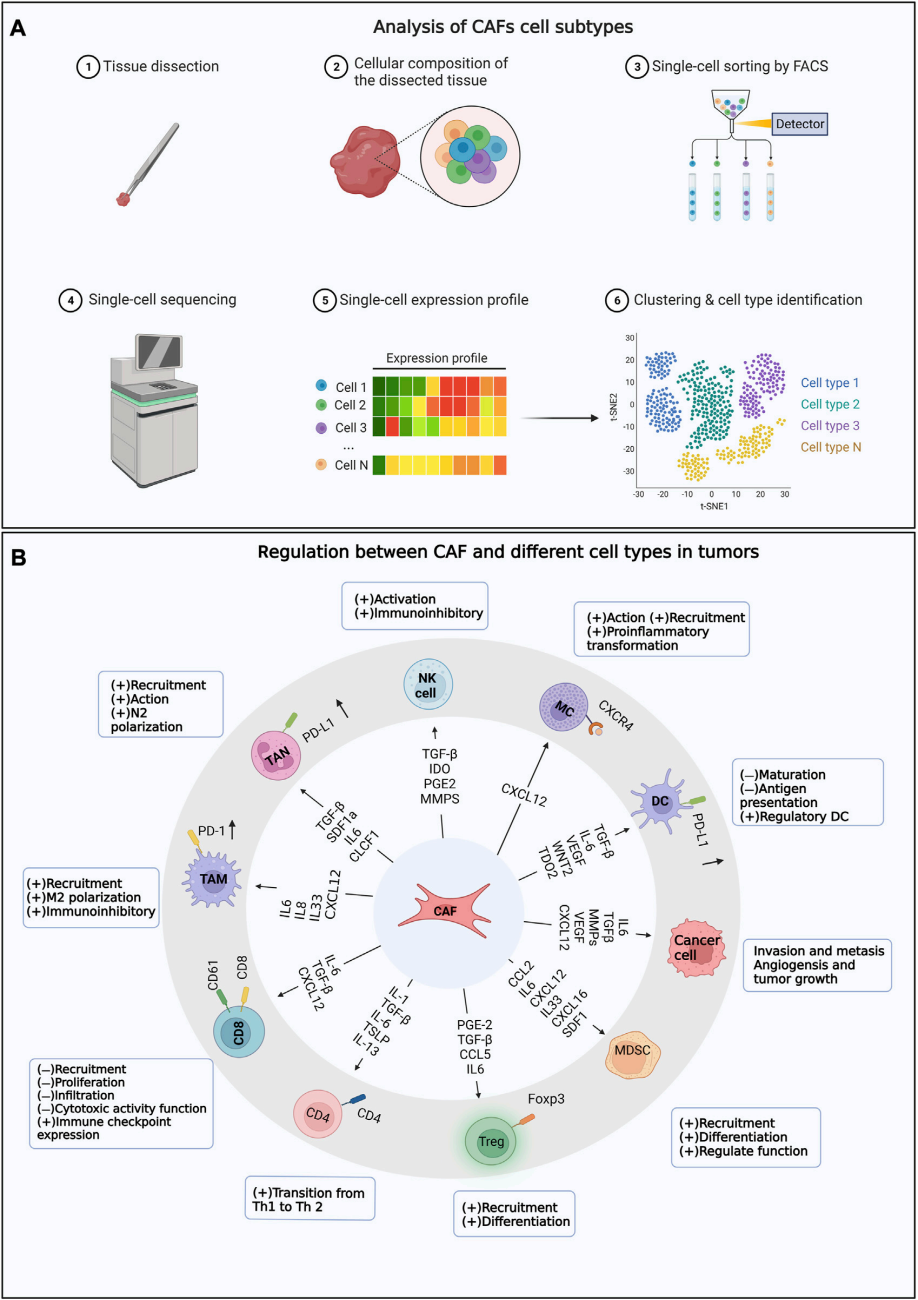
The Research Topic mainly includes the content. **(A)** Analysis of CAFs cell subtypes. **(B)** Regulation between CAF and different cell types in tumors.

In summary, CAFs play crucial roles in tumor progression and treatment. Extensive studies on CAFs facilitate the understanding of tumor biology, identification of new prognostic markers, and development of novel therapeutic strategies as well. However, further researches are necessary to broaden the understanding of CAF functions and regulatory mechanisms for better cancer therapy. The main content of the research topic is shown in Figure 1.

